# Navigating the cohesion-diversity trade-off: understanding the role of facilitators in co-creation using agent-based modelling

**DOI:** 10.1098/rsta.2024.0093

**Published:** 2024-11-13

**Authors:** Sachit Mahajan

**Affiliations:** ^1^ Computational Social Science, ETH Zurich, Stampfenbachstrasse 48, Zurich 8092, Switzerland

**Keywords:** co-creation, facilitation, diversity, cohesion, faultlines, agent-based modelling

## Abstract

In the domain of participatory research and co-creation, understanding the dynamic interplay between group cohesion and diversity is pivotal for fostering innovation. While diversity fuels the generation of novel ideas, cohesion ensures these ideas are effectively synthesized and implemented. This study aims to explore the nuanced role that facilitators play in navigating the balance between cohesion and diversity, particularly in groups characterized by pronounced faultlines. Employing agent-based modeling, the study examines how facilitators affect the cohesion-diversity nexus. The findings reveal a complex facilitator’s paradox. While facilitative actions can successfully enhance group cohesion by mitigating the negative effect of faultlines, such efforts often inadvertently reduce the within-group diversity that is crucial for sparking innovative outcomes. These findings challenge the conventional view of faultlines as merely divisive and underscore the intricate role of facilitators in modulating group dynamics. This research discusses a novel framework for dynamic facilitation strategies, emphasizing the need for facilitators to skillfully balance cohesion and diversity. This framework not only enriches the theoretical understanding of group facilitation but also offers practical insights for optimizing collaborative innovation in diverse settings.

This article is part of the theme issue ‘Co-creating the future: participatory cities and digital governance’.

## 1. Introduction

In the domain of collective intelligence, the ability of collaborative groups to address complex challenges hinges on the interplay between two critical elements: diversity and cohesion. Diversity, encompassing a spectrum of perspectives, experiences and expertise, serves as the engine for innovation, propelling groups towards groundbreaking discoveries and creative solutions [1–3]. Conversely, cohesion fosters a sense of solidarity and shared purpose, underpinning the group’s ability to act harmoniously and execute tasks efficiently [4,5]. The equilibrium between these forces is not only foundational to the success of collaborative endeavours but is also emblematic of the adaptive nature of human collaboration in the face of evolving challenges [6,7]. However, the gravitational pull towards homogeneity can be just as strong as the forces that drive diversification [8,9]. The emergence of ‘echo chambers’, where conformity overshadows dissent, is a testament to the delicate balance that must be navigated to harness the full potential of group collaboration [10,11].

Social and cognitive biases exacerbate the risks associated with homogeneity. In-group bias, a tendency to favour those with perceived similarities, can entrench group dynamics and narrow the ideational bandwidth, restricting the exploration of alternative viewpoints [12,13]. This phenomenon, when coupled with existing divisions within the group, known as faultlines, can further amplify the tendency towards excessive cohesion [14,15]. Faultlines, reflecting potential divisions based on attributes like background, expertise or beliefs, can create subgroups within the larger collaborative environment. Within these subgroups, cohesion might develop rapidly, potentially hindering communication and idea exchange across the wider group [16].

Facilitators are often introduced into collaborative settings to mitigate these challenges. Tasked with fostering inclusivity, mediating conflicts and steering group dynamics, facilitators act as architects of group interaction [17,18]. Despite their pivotal role, there remains a paucity of understanding regarding the facilitator’s ability to navigate the crucial trade-off between cohesion and diversity [19,20]. Achieving an optimal balance is paramount—too little cohesion can fragment a group, while too much can smother the spark of innovation [21].

This study represents an advancement within the domain of co-creation using agent-based modelling (ABM) to explore the intricate dynamics of cohesion, diversity and facilitation within the microcosm of co-creative groups. By simulating the interaction of agents within a controlled virtual environment, this work dissects the multi-layered aspects of group behaviour, exploring the emergent phenomena that arise from the interplay of individual biases, group structures and facilitative interventions. The novelty of the proposed approach lies in its granularity and precision. Through the lens of this modeling approach, the evolution of group dynamics is observed with fidelity capturing the nuances of human interaction. The variables of facilitator effectiveness and cohesion thresholds are adjusted to understand their effect on the equilibrium between diversity and cohesion—a balance that is essential for innovation and effective collaboration.

The findings of this work challenge conventional wisdom, revealing that facilitators, traditionally seen as catalysts for group effectiveness, may inadvertently tip the scales towards excessive cohesion, diminishing the very diversity that is the lifeblood of innovation. This study underscores the importance of a facilitator’s role not just in uniting group members but in creating an environment where diverse ideas merge into a cohesive and dynamic collective intelligence. To address this critical gap, this research investigates the following specific hypotheses:

— *Inherent cohesion hypothesis*: groups with a higher number of faultlines demonstrate an inherent tendency towards high cohesion, challenging the notion that faultlines are purely divisive and suggesting that they may facilitate subgroup cohesion which contributes positively to overall group function.
— *Facilitation and faultline interaction hypothesis*: facilitator interventions, particularly when applied at lower cohesion thresholds, significantly affect group dynamics by reducing faultline strength and inter-group diversity. This suggests a critical facilitator role in managing faultlines to maintain a balance between cohesion and diversity necessary for optimal group performance.
— *Diversity-cohesion equilibrium hypothesis*: there exists an equilibrium state between diversity and cohesion that supports task performance and innovation, which can be disrupted by facilitation. The facilitator’s effect must be carefully modulated to maintain this equilibrium, especially in highly cohesive environments.
— *Facilitator effectiveness paradox hypothesis*: increasing facilitator effectiveness does not linearly improve group outcomes and can lead to diminishing returns. An optimal facilitator effectiveness level is proposed, beyond which the negative effects on diversity outweigh the benefits of increased cohesion.

The research question delves into a nuanced inquiry: how do facilitation interventions affect the balance between cohesion and diversity in co-creation groups and what is the resultant effect on task performance in the presence of faultlines? In advancing our scholarly understanding, this research contributes to the dialogue on collaborative innovation, guiding the management of group dynamics in an era characterized by widespread interconnectedness and collective intellect.

## 2. Background

The exploration of co-creation dynamics within collaborative environments needs a nuanced understanding of the interplay between diversity, cohesion and facilitation. To enrich this understanding, a keyword co-occurrence network analysis was conducted (based on the keywords used in abstracts), drawing on a substantial corpus of literature sourced from the Web of Science spanning from 2015 to 2023. The search query ((‘co-creation’ OR ‘participatory design’) AND ‘facilitat*’) yielded a dataset of 2000 papers, from which a co-occurrence network was created. This analytical approach has been widely used in the identification of dominant themes and the discernment of potential gaps within research areas [22,23]. This section first discusses the current research landscape as revealed by the co-occurrence network analysis, highlighting key clusters and research gaps. Subsequently, it discusses the key theoretical frameworks that underpin this study.

### (a) Current research landscape

**Figure 1.**
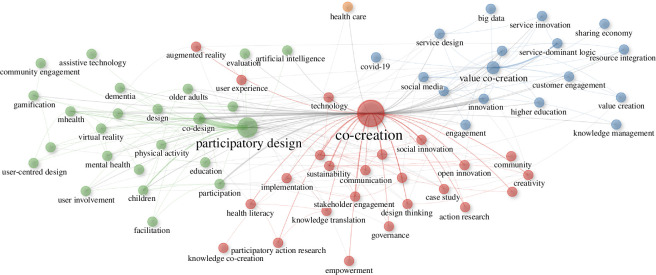
Keyword co-occurrence network mapping the interconnectivity of concepts in participatory and co-creative processes.

Figure 1 shows several clusters where themes like participatory design and value co-creation are prominent. In the participatory design cluster, keywords such as user involvement, co-design and community engagement interact closely with domains like mental health, education and technology, indicating a robust emphasis on user-centric methodologies in diverse settings. Similarly, the value co-creation cluster incorporates elements such as stakeholder engagement and social innovation, highlighting its application in broader contexts including business and governance. Despite these rich thematic interactions, facilitation—a key element in managing and guiding these complex interactions—remains conspicuously under-represented in the literature. Effective facilitation is crucial in ensuring that the diverse inputs in participatory settings are synthesized effectively, yet it rarely surfaces as a central theme of investigation. This oversight is particularly striking given the intricate dynamics within clusters where multiple stakeholders must align their varied interests and perspectives.

### (b) Theoretical frameworks and concepts

Co-creation, as a concept, has evolved significantly over the past few decades. Historically rooted in the participatory design movements of the 1970s, co-creation has expanded across various fields, including business, urban planning and healthcare. The theoretical underpinnings of co-creation are deeply influenced by Vygotsky’s socio-cultural theory, which emphasizes the importance of social interaction in cognitive development [24]. This perspective aligns with the collaborative nature of co-creation, where knowledge is constructed through shared experiences and dialogue.

Diversity has long been lauded for its contribution to enhancing problem-solving capabilities and fostering innovation within groups [25,26]. The co-occurrence network graph (figure 1) echoes these sentiments, with nodes like ‘innovation’, ‘co-creation’ and ‘co-design’ forming a dense network, reflecting the vibrancy of diversity’s role as recognized by Hong and Page [27]. Yet, the network analysis has brought to light a pivotal research gap—the peripheral placement of ‘facilitation’ within the participatory design cluster. This gap underscores a potential oversight in the literature where the symbiosis of facilitation with the mechanisms of diversity has not been deeply probed.

Cohesion’s importance has been widely discussed in the area of group dynamics, where it underpins mutual respect and collective efficacy. Excessive cohesion, however, can lead to conformity that dampens critical thinking [28,29]. The node ‘value co-creation’ emerges as a significant cluster related to cohesion, hinting at the coalescence of diverse stakeholders around shared value creation, a concept well-articulated in the works of Vargo and Lusch [30]. Nevertheless, the weak links to facilitation imply a research gap. The literature acknowledges the importance of cohesion but seldom explores the granularity of how facilitation can navigate its complexities without constricting cognitive diversity.

Facilitation stands at the confluence of diversity and cohesion, steering group processes and harmonizing disparate perspectives [31–33]. Its capacity to influence group outcomes is acknowledged yet not exhaustively explored within co-creative contexts [17]. The network graph illustrates that while facilitation is integral to participatory practices, it exhibits a lower centrality in the network, suggesting that its strategic role in navigating diversity and cohesion is less explored. Conway’s Law [34] also offers an insight here, suggesting that the structure of communication within groups influences the outcomes of co-creation, thereby underlining the importance of facilitation in shaping effective communication pathways. Moreover, the concept of ‘emergence rather than extraction’, discussed by Ardoin *et al*. [35], suggests that facilitation should focus on fostering conditions for the organic emergence of ideas and solutions rather than extracting predefined results. This aligns with participatory research principles, advocating for environments that nurture natural innovation through dynamic interactions. This approach resonates with the broader goals of co-creation, emphasizing the importance of creating adaptive and responsive group dynamics that can evolve organically.

In citizen science, the combination of diverse non-professional contributions into a cohesive research output is critical for success [36–38]. It often needs effective facilitation strategies that can maintain the richness of diverse contributions within an organized framework. Similarly, in participatory city-making [11], the move beyond inclusion towards leveraging diversity [39] demands facilitation that ensures diverse voices are not just heard but integrated into decision-making processes. This integration supports the successful transformation of urban spaces and aligns with recent findings on participatory planning [40,41]. The role of facilitation emerges as crucial in bridging gaps between diverse stakeholders, playing a pivotal role in the ecosystem of participatory processes by nurturing a collaborative environment where a multitude of perspectives can converge to drive innovation. The facilitator’s role transcends process guidance, extending to the modulation of group dynamics to foster environments where diversity is harnessed effectively, an aspect explored by Ansell and Gash [18] in the context of collaborative governance.

### (c) Bridging theory and evidence

ABM offers a powerful framework for simulating and analysing the intricate dynamics of interaction in social settings [42]. ABM provides an experimental ground to validate the theoretical constructs of diversity, cohesion and facilitation within controlled scenarios. This approach is informed by complex adaptive systems (CAS) theory, which suggests that groups and organizations are complex systems that adapt and evolve in response to internal and external stimuli [43]. Pioneering studies, such as those by Axelrod [44] and Tornberg [45], have demonstrated the utility of ABM in exploring how cultural dissemination and group polarization evolve through localized interactions. Axelrod’s model highlights how local convergence of cultural traits can lead to global polarization. Similarly, Tornberg’s research into the dynamics of polarization within groups reveals the conditions under which group consensus and division emerge, emphasizing the role of homophily and social influence. These models underscore the responsiveness of ABM to subtle changes in agent behaviours and interaction rules, making them ideal for studying the nuanced dynamics of group cohesion and diversity. Furthermore, the work by Mäs [46] discusses how faultlines across multiple demographic dimensions can exacerbate polarization. These studies collectively form a substantial foundation for understanding the intricate dynamics of social interactions within groups, which are critical to the study of co-creation.

Despite the rich insights provided by existing ABM research into social interactions and group dynamics, there remains a significant gap in models specifically designed to assess the effect of facilitation on co-creation processes. Facilitation, particularly in co-creative settings, involves not just the enhancement of group interaction but also the strategic alignment of these interactions towards creative and productive outcomes. This research aims to bridge this gap by extending traditional ABM approaches to explicitly model facilitation’s role within co-creative processes.

## 3. Methodology

This study utilizes ABM to dissect the intricate dynamics of knowledge creation within groups, particularly focusing on the roles of diversity, cohesion and facilitation. ABM is ideal for this research owing to its ability to capture emergent behaviours from complex interactions among individual agents [47,48].

### (a) Agent description

The model proposed in this work comprises a set of N agents, each characterized by distinct attributes that simulate individual differences within a collaborative group. These attributes are:

— *Attribute vector*: similar to the model proposed by Axelrod [44], each agent i is assigned an attribute vector $a_{i}=(a_{i1}{,a}_{i2},\ldots ,a_{im})$ , where m denotes the number of attributes per agent and each attribute $a_{ij}$ can assume one of M possible values, representing the variety of skills, knowledge or opinions the agent contributes to the group.
— *Openness to facilitation*: this is represented by $o_{i}$ , a continuous value ranging from 0 (completely resistant to facilitation) to 1 (fully open to facilitation). This measure influences the likelihood of an agent altering its attributes in response to the facilitator’s actions. The conceptualization of this attribute is informed by existing literature [49,50] that explores the dynamics of agent responsiveness in simulated environments.
— *Initial faultline*: each agent is initially assigned to a faultline $f_{i}$ where $f_{i}\in \{1,2,\ldots ,F\}$ and F signifies the total number of distinct faultlines identified within the group. These faultlines symbolize potential internal divisions that could affect the group’s dynamics and cohesion [51].

The model progresses in discrete time steps, with the following key processes occurring at each step:

1. *Agent interactions*: at every time step, each agent i selects a subset of other agents to interact with, determined by a parameter *γ *, representing the agent’s propensity to engage with others [45,52]. To mimic realistic social constraints, the number of interactions any agent can initiate per time step is capped at a maximum of 5. This ensures a manageable level of engagement reflective of limited attention spans and interaction capabilities in real-world settings.
2. *Similarity calculation*: the foundation of interaction lies in the perceived similarity between agents [53]. The similarity score $s_{ij}$ between agents i and j is computed based on their attribute vectors, using the formula
$$s_{ij}=\;\frac{1}{m}\;\sum_{k=1}^{m}\delta \left(a_{ik}\;,\;a_{jk}\right),$$
  where $\delta (a_{ik},a_{jk})$ is a binary indicator function, defined as 1 if $a_{ik}=a_{jk}$ (i.e. the attributes match) and 0 otherwise. This formula computes the average number of matching attributes between the two agents.
3. *Interaction probability*: the probability $P_{ij}$ of agent i interacting with agent j is determined by their similarity and the sensitivity parameter h , according to
$$P_{ij}=\;\frac{\exp\left(h\cdot s_{ij}\right)}{\sum_{k\neq i}\exp\left(h\cdot s_{ik}\right)}.$$
4. *Attribute Update*: following an interaction, agent i has an opportunity to update one of its attributes to match that of the interacting agent j , with the updated probability influenced by $o_{i}$ . This process of attribute adjustment is informed by the principles outlined in the work by Axelrod [44].

### (b) Group cohesion calculation

Group cohesion is quantified by assessing the average similarity within faultline groups [15], encapsulating the degree of alignment and shared understanding among group members. Mathematically, this is expressed as


$$Group\;\;cohesion=\;\frac{1}{F}\sum_{f=1}^{F}\left(\frac{2}{n_{f\;}\left(n_{f}-1\right)}\;\sum_{i=1}^{n_{f}}\sum_{j=i+1}^{n_{f}}s_{ij}\right),$$

where F is the total number of faultlines, $n_{f}$ is the number of agents within a given faultline group f and $s_{ij}$ is the similarity score between agents i and j .

### (c) Inter-group diversity calculation


$$Inter\;-\;group\;diversity=\;\frac{1}{\left|S\right|}\sum_{\left(a,b\right)\in \;S}\left(1-\;s_{ab}\right),$$

where S is the set of all pairs ( a,b ) such that agents a and b belong to different faultline groups ( $f_{a}\neq f_{b}$ ); $\left|S\right|$ represents the cardinality of set S , $s_{ab}$ is the similarity score between agents a and b , with $1-s_{ab}$ quantifying their attribute dissimilarity.

### (d) Task simulation

Agents collaborate on a task requiring a blend of knowledge diversity and cohesion. Task performance T at each time step is evaluated as


$$T=\left(\frac{Inter\;-\;group\;diversity+Group\;cohesion}{2}\right)\times \;\left(1-C\right),$$

where C represents task complexity, emphasizing the dual importance of diversity and cohesion in achieving success.

### (e) Facilitator introduction

A facilitator is introduced into the model with an effectiveness parameter e , where e∈[0,1] . The facilitator’s actions are designed to influence group dynamics through the following mechanisms:

1. *Promoting common goals*: increases the similarity threshold, encouraging agents to find common ground.
2. *Encouraging interactions*: boosts γ , promoting interactions across faultlines, thereby facilitating broader communication within the group.
3. *Adaptive interventions for empathy and diversity*: the facilitator’s approach adapts based on real-time group metrics:
  — *Fostering empathy*: targeted when inter-group diversity falls below a critical threshold (e.g. 0.4), this action aims to deepen understanding and appreciation of diverse perspectives, mitigating the risks of echo chambers.
  — *Enhancing diversity*: activated if within-group diversity drops beneath a specified level (e.g. 0.3), the facilitator intervenes to diversify attributes within homogenous clusters, promoting a richer spectrum of ideas and viewpoints.

These context-sensitive actions ensure that facilitation is not only strategic but also responsive to the evolving landscape of group dynamics, with effectiveness finely tuned by the parameter e and individual agents' openness to change $o_{i}$ .

### (f) Gradual influence mechanism

Reflecting a nuanced understanding of group evolution, the facilitator’s influence progressively intensifies over time. With each modeling step, the facilitator’s effect incrementally strengthens, simulating the growing trust and authority a facilitator might naturally have within a group. This gradual approach allows for a more organic integration of facilitative actions, mirroring the subtleties of real-world group facilitation [54,55]. Facilitator interventions are activated when group cohesion surpasses a predefined threshold $T_{c}$ , highlighting scenarios where excessive cohesion might hinder creativity and innovation.

The model tracks various metrics to analyse system dynamics and the effects of interventions:

— *Group cohesion*: calculated as the average similarity within each faultline group.
— *Inter-group diversity*: assesses the average dissimilarity of attributes across different faultline groups.
— *Faultline strength*: quantified through the average entropy [56] of attribute value distributions within each faultline group, serving as an indicator of internal heterogeneity. This measure reflects the diversity or uniformity of agent attributes within groups, which can influence group dynamics and performance. Entropy ( $H_{f}$ ) for faultline group f is calculated as

$$H_{f}=\;-\sum_{i=1}^{n}p_{i}\;log_{2}\left(p_{i}\right),$$

where $p_{i}$ represents the probability of each unique attribute value combination, and *n* is the total number of unique combinations.

— *Within-group diversity*: average attribute dissimilarity among agents within the same faultline group.

### (g) Simulation phases

This study is structured into two phases to systematically explore the effect of facilitator interventions under varying conditions of initial group cohesion and facilitator effectiveness. Prior to the detailed exploration of group dynamics, a sensitivity analysis was performed to identify key optimal parameters for γ (interaction propensity) and h (homophily), crucial for modeling agent interactions and subgroup formation. For γ , values ranging from 0 to 1 were tested to assess their effect on group cohesion, inter-group diversity and task performance. Similarly, sensitivity analysis for h explored three distinct levels: low [1], medium [4] and high [8], following the categorization explored by Tornberg *et al*. [45].

In phase 1, the model operates without the influence of a facilitator to establish a baseline understanding of group dynamics across varying numbers of faultlines. This phase aims to capture the inherent system dynamics under different structural conditions, providing insights into how diversity and cohesion naturally evolve within the group. The primary focus is to examine:

— How faultlines affect group cohesion and diversity without external interventions.
— The effect of inherent group structures on task performance.

Phase 2 introduces the facilitator into the modeling environment, with a focus on understanding how targeted interventions can modulate group dynamics to enhance collaborative outcomes. Simulations are conducted under a range of cohesion thresholds $\left(T_{c}\right)$ and facilitator effectiveness levels (e) to explore:

— The effect of facilitation at varying levels of group cohesion and the effectiveness of facilitation strategies in promoting optimal balance between cohesion and diversity for task success.
— The role of the facilitator in managing faultlines.

Throughout both phases, the model iteratively advances through set time steps, facilitating the observation of dynamic shifts in group behaviours and performance over time. This iterative, time-step-based approach enables a comprehensive examination of the temporal dynamics of group interactions and the longitudinal influence of facilitation efforts.

To ensure fidelity to the complex nature of group dynamics, the methodology incorporates several key mathematical formulations and assumptions:

— *Dynamic interaction model*: the interaction probability ( $P_{ij}$ ) and attribute update mechanisms are designed to reflect the fluidity of real-world group interactions, where individuals continuously adapt and evolve based on their experiences and exposures within the group.
— *Facilitation modulation*: the facilitator’s actions—ranging from promoting common goals to fostering diversity—are quantified through probabilistic interventions that mirror the nuanced role facilitators play in guiding group dynamics towards productive outcomes.
— *Complexity adjustment*: the task performance equation incorporates a complexity parameter C , acknowledging that the nature of the task itself significantly influences the requisite balance between diversity and cohesion for optimal group performance.
— *Threshold-driven interventions*: the use of a cohesion threshold $\left(T_{c}\right)$ for triggering facilitator interventions underscores the model’s responsiveness to the evolving state of the group, allowing for a more tailored and effective facilitation approach.

## 4. Results

This study commenced with a carefully structured model designed to investigate the fundamental dynamics of group behaviour in the absence of facilitation. An ABM was created to simulate the interactions and evolution of group dynamics over a series of discrete time steps. This phase was pivotal in establishing a baseline against which the influence of facilitation could be measured. In addition to the ABM, the analytical approach was designed to provide a robust statistical understanding of group dynamics. To achieve this, both the mean (M) and standard deviation (SD) of the outcomes across various metrics were examined, supplemented by *t*-tests. These tests evaluated the significance of the observations, comparing the mean of a sample to a known value to determine if the differences observed are statistically significant.

**Figure 2.**
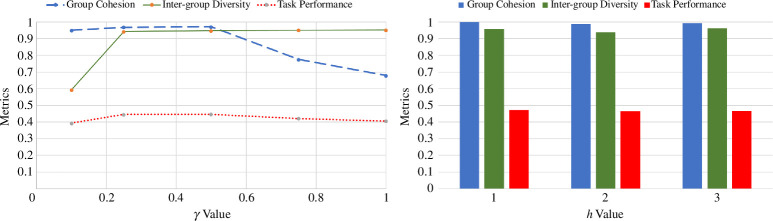
Sensitivity analysis of group dynamics metrics. The left graph displays the relationship between the γ (gamma) value and three key metrics: group cohesion, inter-group diversity and task performance. The right graph illustrates the effect of varying levels of homophily (h) on the same metrics, represented through a bar chart.

The sensitivity analysis, conducted to determine optimal settings for key model parameters γ (interaction propensity) and *h* (homophily) yielded insights into their effects on group cohesion, inter-group diversity and task performance. As shown in figure 2, a γ value of 0.5 emerged as notably effective, striking a balance between maintaining high group cohesion, inter-group diversity and achieving a high task performance observed across the range. Similarly, for *h*, a low value [1] supports an optimal balance with perfect group cohesion (1.0), high inter-group diversity (0.957) and highest task performance (0.457) as compared to other scenarios.

The model involved 50 agents—each provided with an attribute vector comprising five attributes. These attributes could adopt 1 of 10 possible values, representing the diverse characteristics and perspectives each agent brought to the group. To explore the inherent dynamics of these virtual groups, structural settings were varied by implementing faultline levels set at two, four and six. These faultline configurations served as a proxy for potential divisions within the group, based on the similarity of agent attributes. The faultline parameter was central to understanding how subgroup formation might affect overall group cohesion and performance—a core focus of this research.

### (a) Phase 1: Baseline group dynamics

Each simulation ran for 500 iterations to ensure the stability of emergent patterns and was repeated over 20 independent runs to guarantee the robustness of the findings. During these iterations, agents engaged in interactions determined by an interaction propensity γ=0.5 , influenced by the strength of their attribute similarities. The sensitivity to attribute similarity was moderated by a homophily parameter *h* = 1.0, reflecting the tendency of agents to engage with those possessing like attributes. A task complexity parameter (*c* = 0.5) was also included to simulate the challenges inherent in collaborative tasks and their requirements for both diversity and cohesion for successful completion.

#### (i) Inter-group diversity and faultline dynamics

**Figure 3.**
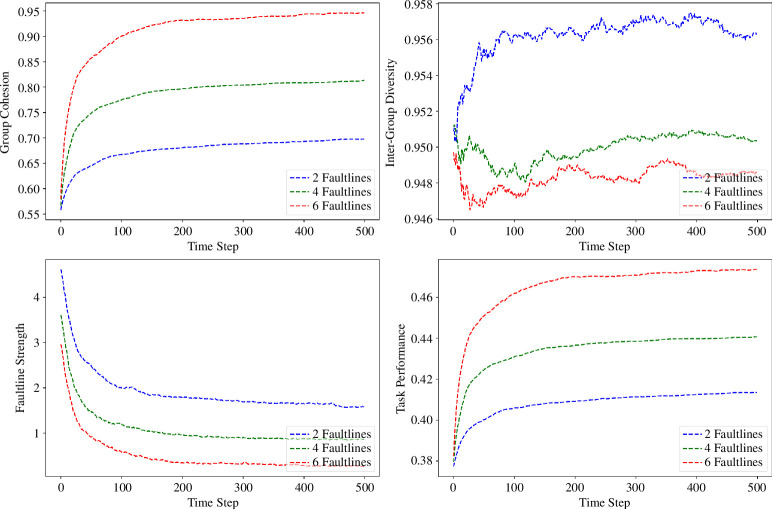
The line graphs show the evolution of group cohesion (top left), inter-group diversity (top right), faultline strength (bottom left) and task performance (bottom right) for different faultline structures. Each line represents the mean trajectory of 20 simulation runs.

Contrary to the potential for fragmentation, inter-group diversity remains robust across the faultline variations, with statistical tests yielding highly significant results (two faultlines: *t*-statistic = 201.464, *p*‐value = 0.0001). This finding suggests that groups are naturally inclined to maintain a diversity of attributes, which is critical for collective problem-solving and innovation. An inverse relationship is seen in faultline strength, where an initial high *M* (1.577) for two faultlines significantly decreases (*t*-statistic = 28.477, *p*‐value = 0.0001) as the number of faultlines increases. This trend underscores the diffusion of subgroup identities, contributing to a more integrated group structure over time. The nonlinear relationship between faultline strength and the number of faultlines indicates complex interaction dynamics that do not simply linearly increase or decrease but instead show varied patterns of influence depending on the specific configuration of faultlines. Task performance consistently improves with the complexity of faultlines. The six-faultline configuration exhibits a notable performance *M* of 0.474 (SD = 0.009), supported by a significant *t*-statistic (228.098), suggesting that cohesive subgroups formed along faultlines can collectively enhance the group’s operational effectiveness. The results (figure 3) show an interesting trend: groups inherently trend towards cohesion, with six-faultline configurations showing the highest value (*M* = 0.946, SD = 0.033). This observation is statistically significant (*t*-statistic = 125.357, *p*‐value = 0.0005), affirming the hypothesis that increased faultlines may not be as divisive as traditionally believed, but rather can enhance unity within the group.

### (b) Phase 2: Facilitation impact analysis

In phase 2, the objective was to understand the effect of a facilitator’s interventions on group dynamics at varying levels of inherent group cohesion. A facilitator was integrated into the ABM, capable of influencing group cohesion and diversity across 500 iterations and 20 runs. This phase focused on a six-faultline group structure, identified in phase 1 as critical, at two cohesion thresholds—0.85 and 0.4—to reflect different facilitator intervention strategies to optimize group innovation and task performance.

### (c) Facilitation at cohesion threshold of 0.85

**Figure 4.**
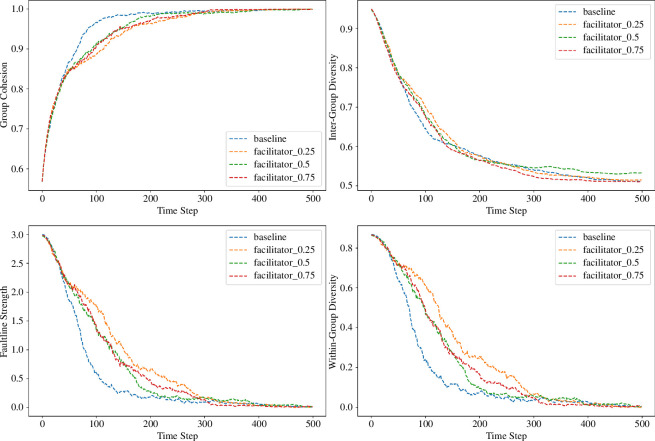
Dynamic trends of group cohesion, inter-group diversity, faultline strength and within-group diversity across different facilitation intensities. The figure depicts the simulation results at a cohesion threshold of 0.85, demonstrating the effects of facilitator interventions over time.

At a high cohesion threshold of 0.85, facilitator interventions were strategically implemented to mitigate the risk of excessive cohesion within groups. Figure 4 illustrates the dynamic trends across key metrics in varying facilitation conditions.

The empirical data (table 1) support the premise that facilitators play a pivotal role in unifying groups, as evidenced by the maintained high levels of group cohesion across all facilitation scenarios (*M* ≈ 1.000). It is crucial to note, however, that while the absolute changes in group cohesion are subtle, the stability of cohesion maintained across various conditions underscores the effectiveness of facilitation in ensuring consistent group performance and harmony. This role extends beyond simply maintaining high cohesion. Facilitators help sustain these high levels consistently across different scenarios, ensuring stability in group cohesion, which is essential for effective collaboration.

**Table 1.** Group dynamic metrics (for $T_{c}$ = 0.85) under different facilitation conditions compared to the baseline.

condition	group cohesion	inter-group diversity	faultline strength	task performance	within-group diversity
baseline	1.000 (±0.002)	0.511 (±0.040)	0.007 (±0.030)	0.378 (±0.010)	0.003 (±0.014)
facilitator 0.25	1.000 (±0.001)	0.515 (±0.065)	0.003 (±0.014)	0.379 (±0.016)	0.001 (±0.005)
facilitator 0.5	1.000 (±0.001)	0.533 (±0.082)	0.010 (±0.031)	0.383 (±0.020)	0.004 (±0.011)
facilitator 0.75	0.999 (±0.006)	0.511 (±0.037)	0.019 (±0.083)	0.377 (±0.008)	0.007 (±0.031)

A nuanced analysis of the inter-group diversity metric reveals a complex picture. Contrary to an expected decline, the inter-group diversity remained relatively stable across the facilitation conditions compared to the baseline (*M* = 0.511, SD = 0.040 for the baseline and *M* = 0.511, SD = 0.037 for the highest facilitation condition). This finding suggests that while facilitators contribute to cohesion, they do not necessarily diminish the diversity among different subgroups within the group, which is vital for fostering innovative and effective solutions. The dynamics of faultline strength are particularly informative. While the empirical results indicate some variability, the 0.25 facilitation effectiveness condition shows a notable decrease in faultline strength (*M* = 0.003, SD = 0.014), suggesting that even a modest level of facilitator intervention can significantly bridge subgroup divisions. However, it is noted that as facilitation levels increase, there is a nonlinear response in faultline strength, which does not always correlate with higher levels of facilitation, leading to lower faultline strengths. This complexity highlights the nuanced and sometimes unpredictable effects of facilitation, which may vary depending on the specific dynamics and existing tensions within the group. Task performance slightly increased with facilitator presence (*M* = 0.379, SD = 0.016 at 0.25 facilitation effectiveness), hinting at a possible enhancement in the group’s problem-solving capabilities with facilitation, albeit marginally. This observation suggests that while facilitation can enhance certain aspects of group dynamics, its effect on task performance is not linear and may be influenced by a variety of factors including the initial conditions of the group and the specific nature of the tasks involved.

Nevertheless, this increase is not consistent across all facilitator conditions, indicating that the relationship between facilitator interventions and task performance might be nonlinear or influenced by other moderating factors. Within-group diversity exhibited a decline across all conditions, including the baseline. However, facilitation appears to mitigate this decline to varying degrees. The most effective facilitator condition (0.75 effectiveness) showed a higher within-group diversity (*M* = 0.007, SD = 0.031) compared to the baseline (*M* = 0.003, SD = 0.014), suggesting that skilled facilitation can maintain a more heterogeneous subgroup composition. This indicates that facilitators help preserve diversity within subgroups, which is crucial for fostering a rich cognitive resource pool and enhancing creative problem-solving abilities.

### (d) Facilitation at cohesion threshold of 0.4

**Figure 5.**
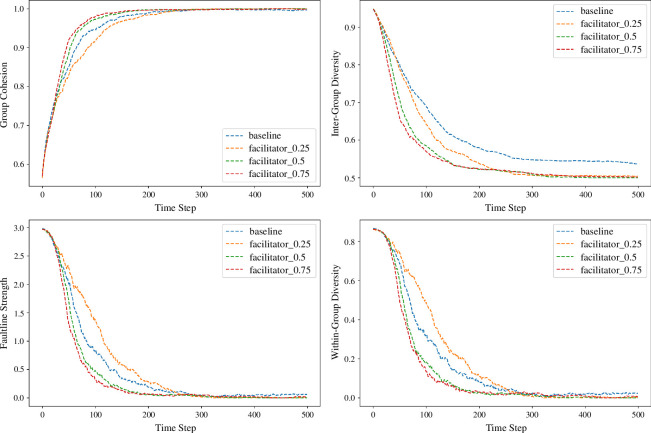
The line graph represents the evolution of four key group metrics at a cohesion threshold = 0.4: group cohesion, inter-group diversity, faultline strength and within-group diversity across several iterations. Each metric is plotted for various facilitator effectiveness levels (0.25, 0.5, 0.75) alongside a baseline scenario with no facilitator.

After adjusting the cohesion threshold to 0.4, the objective was to promote a balanced level of diversity while maintaining group unity. The results, depicted in figure 5 and listed in table 2, illustrate the effects of facilitation at this lower threshold.

**Table 2.** Group dynamic metrics (for $T_{c}$ = 0.4) under different facilitation conditions compared to the baseline.

condition	group cohesion	inter-group diversity	faultline strength	task performance	within-group diversity
baseline	0.997 (±0.008)	0.537 (±0.072)	0.063 (±0.141)	0.383 (±0.017)	0.023 (±0.051)
facilitator 0.25	1.000 (±0.001)	0.505 (±0.014)	0.006 (±0.028)	0.376 (±0.003)	0.003 (±0.013)
facilitator 0.5	1.000 (±0.001)	0.500 (±0.054)	0.003 (±0.006)	0.375 (±0.001)	0.001 (±0.002)
facilitator 0.75	0.999 (±0.004)	0.502 (±0.009)	0.016 (±0.071)	0.375 (±0.001)	0.007 (±0.031)

Group cohesion was consistent across all facilitation conditions, mirroring the trends observed at the higher cohesion threshold of 0.85. This constancy highlights the effectiveness of facilitation strategies in unifying group members across different initial cohesion levels. Comparatively, the facilitator’s influence in maintaining cohesion is seemingly unaffected by the threshold alteration, reinforcing the facilitator’s substantial role in group dynamics. At both the 0.85 and 0.4 thresholds, facilitated conditions yielded similarly high cohesion levels, indicating a resilience of facilitator impact against varying initial group cohesiveness. Regarding subgroup faultlines, the facilitated conditions, particularly at the initial stages, demonstrated a substantial reduction in faultline strength, converging on a plateau thereafter. This trend aligns with the observations at the higher threshold but is notably more pronounced in the current scenario. The empirical evidence suggests that facilitators are particularly effective in contexts of lower initial group cohesion, where their interventions can enhance group unity.

The decline in within-group diversity was more pronounced at the lower threshold (*M* = 0.007, SD = 0.031 at the highest facilitation effectiveness), suggesting that the facilitation may inadvertently standardize subgroup characteristics, potentially at the expense of diversity. This outcome could be indicative of the facilitator’s effectiveness in promoting common ground at the potential cost of reducing subgroup variance.

However, it is essential to consider the trade-offs between achieving high cohesion and maintaining diversity. The task performance metrics did not exhibit a clear benefit from increased cohesion or reduced faultlines, as evidenced by the slight underperformance across varying facilitation levels compared to the baseline. This trend suggests that the relationship between cohesion and task efficiency is complex and nonlinear, with facilitation playing a role in optimizing this balance. Furthermore, inter-group diversity showed a modest decrement across facilitation levels, albeit to a lesser extent than at the higher threshold. This could suggest a more nuanced interaction between facilitator actions and inherent group diversity dynamics with facilitation exerting an influence that, while still noticeable, is less dramatic under the conditions of the lower threshold.

In summary, the outcomes from phase 2 underscore the multifaceted influence of facilitators on group cohesion and the complexities in safeguarding diversity. The consistent achievement of high group cohesion across varying thresholds and facilitation intensities speaks to the effectiveness of facilitators in uniting group members. However, the simultaneous decline in both within-group and inter-group diversity signals a potential challenge for facilitators: the risk of homogenizing group perspectives, which can be counterproductive to innovation. The slight underperformance in task execution across facilitation conditions, relative to the baseline, further reinforces the intricate balance between cohesion and diversity. These empirical insights underscore the importance of developing facilitation strategies that optimize group dynamics, promoting cohesion while preserving the diversity essential for fostering creative problem-solving and ensuring task efficacy. Understanding these dynamics is imperative for devising facilitator interventions that support a group environment conducive to sustained innovation and performance.

## 5. Discussion and conclusion

This exploratory study examined the intricate dynamics of group behaviour in co-creation contexts, particularly focusing on the dual forces of cohesion and diversity and how they are modulated by facilitation. The ABM, designed to mimic real-world participatory group interactions, unearthed several counterintuitive phenomena that challenge established beliefs in the field of collaborative innovation.

### (a) Cohesion and facilitation dynamics

Phase 1 of the study highlighted the natural trajectory of groups towards cohesion, lending robust support to the inherent cohesion hypothesis. The findings of phase 1 revealed that faultlines did not inherently fragment groups but rather contributed to subgroup cohesion, which in turn strengthened overall group integrity. This emergent cohesion was statistically significant, especially in groups with a greater number of faultlines, suggesting that under certain conditions, structural divisions might serve as a scaffold for collaboration rather than a barrier. As the study transitioned to phase 2, the introduction of a facilitator was observed to further amplify cohesion, aligning with the established objectives of facilitation in participatory research. However, the simulation results demonstrated that heightened cohesion, particularly when pushed by the facilitator’s actions, was accompanied by a marked decrease in diversity. This decline spanned both inter-group and within-group diversity and was most notable in scenarios of higher facilitator effectiveness. The nonlinear response of faultline strength to facilitation effectiveness highlights the complexity of these dynamics, indicating that increased facilitation does not always linearly correlate with decreased faultline strength.

### (b) Balancing act: cohesion, diversity and group functioning

The observed trade-off between cohesion and diversity informs critical considerations for facilitation in group settings. Cohesion undoubtedly enhances group collaboration, but it also implies that, without careful management, it might reduce the diversity of thought, which is often associated with innovative potential. Although not directly measured in this study, the theoretical link between diversity and innovation suggests that an overemphasis on cohesion could potentially limit the group’s innovative capacity. Facilitators, therefore, face the complex task of modulating their strategies to support both cohesion and the retention of diverse perspectives that drive innovation. This nonlinear and dynamic relationship underscores the need for facilitators to adapt their interventions based on real-time feedback, ensuring that the balance between cohesion and diversity is maintained. Exploring real-time feedback mechanisms and decision-making tools may prove beneficial in achieving this balance, warranting further investigation. These interventions could provide facilitators with dynamic measures to adjust their approach in accordance with the group’s changing needs, potentially preserving the essential diversity that contributes to robust problem-solving and idea generation.

### (c) Practical implications and future directions

The findings of this study have significant implications for citizen science, co-creative processes and participatory research. In these contexts, facilitation is a critical factor for the successful integration of diverse knowledge bases. This study underscores the necessity for facilitators to not only achieve group consensus but also to actively promote and preserve diversity within the group. This approach is crucial as diversity is traditionally associated with creative and innovative outcomes. Looking ahead, it would be valuable to test the model with groups of different sizes and within various cultural contexts. Such studies would enhance our understanding of the generalizability of our findings and inform the adaptation of facilitation techniques to diverse group dynamics.

### (d) Reflective conclusions

As the study concludes, it is important to reflect on the vital role of social dynamics in collaborative innovation. The results of this study shed light on the subtle yet profound influence that facilitators wield within group settings. Although the model has provided valuable insights, it also underscores the inherent limitations of simulations, which cannot fully capture the multifaceted nature of human interaction and decision-making. Simulations, by necessity, simplify real-world complexities to create manageable and interpretable models. This simplification means that certain nuances and unpredictable human behaviours may not be fully represented. The model relies on several key assumptions and parameters, such as the levels of agent interaction propensity and homophily, which are based on empirical data and theoretical considerations. However, the sensitivity of the outcomes to these parameters highlights the need for careful calibration and validation against real-world data.

This research emphasizes the importance for facilitators to be aware of the delicate impact their interventions may have on the balance between group cohesion and diversity. Further empirical research is essential to refine our understanding of these dynamics. The ultimate aspiration is to leverage the collective capability of groups in co-creation settings, striving for innovation that is both inclusive and effective. This study contributes to the ongoing discourse on group dynamics, advocating for an evolved facilitation approach that honours and harnesses the diversity fundamental to innovation and progress in collaborative tasks.

## Data Availability

The code supporting the findings of this study has been deposited in Zenodo [57].
